# Replicating research in ecology and evolution: feasibility, incentives, and the cost-benefit conundrum

**DOI:** 10.1186/s12915-015-0196-3

**Published:** 2015-10-28

**Authors:** Shinichi Nakagawa, Timothy H. Parker

**Affiliations:** School of Biological, Earth and Environmental Sciences, University of New South Wales, Sydney, NSW 2052 Australia; Biology Department, Whitman College, Walla Walla, WA 99362 USA

## Abstract

We believe that replicating studies in ecology and evolution is extremely valuable, but replication within species and systems is troublingly rare, and even ‘quasi-replications’ in different systems are often insufficient. We make a case for supporting multiple types of replications and point out that the current incentive structure needs to change if ecologists and evolutionary biologist are to value scientific replication sufficiently.

## The foundation of cumulative science

Science is largely a cumulative process of building upon previous findings. For this process to work, we must assume that previous scientific findings are real and replicable. Dismayingly, this fundamental assumption may often be incorrect. Failures of reproducibility in medical and social sciences have received considerable attention recently [1, 2], in part because some of these studies had important implications for human health and society. In response, high profile replication efforts are currently underway, most notably in psychology (for example, [3, 4]). However, the extent to which the disciplines of evolutionary biology and ecology will embrace replication remains to be seen.

In the fields of ecology and evolution (in which we work), faithful replication is typically difficult because we study a diverse array of species in a range of often variable settings. Such difficulties, however, do not diminish the importance of reproducibility. Two recent meta-analyses of well-studied systems (zebra finch, *Taeniopygia guttata* [5] and blue tit, *Cyanistes caeruleus* [6]) revealed that positive findings may often be either laboratory/population-specific, or even more problematically, due to Type I error. The former meta-analysis, based largely on a series of replications carried out by the authors, soundly contradicted previous findings that applying red colour rings to male zebra finches increased both their courtship behaviour and body weight, thus enhancing their attractiveness. The latter meta-analysis found evidence of many practices that inflate error. Further, despite the publication of dozens of studies of blue tit colour and many hundreds of statistical effects, the rarity of actual replication in the literature left insufficient evidence to support most of the frequently cited claims regarding the sexually selected role of plumage colour in this species. These meta-analyses are not the first to draw such sobering conclusions in our field [7–9]. Low reproducibility has been recognized as an issue in ecology and evolution for a long time, but little has been done to confront it.

Here, we reinforce and expand upon previous critiques of replication in ecology and evolution going back many years [9, 10]. We aim to review major issues and to encourage constructive thinking about a future for replication in our disciplines. We finish by discussing some specific initiatives, some of which have already been initiated in social and medical sciences (for example, [11]), to encourage research replications.

## What role should replication play in evolution and ecology?

We believe that replication holds tremendous potential for promoting scientific progress, though we recognize that not all work in our disciplines can be replicated and that not all ecologists and evolutionary biologists agree that the benefits of replication outweigh the costs [9]. We replicate for two reasons: (i) to assess the validity of prior findings, and (ii) to probe the generality of those findings. In ecology and evolutionary biology, we often do quite a poor job proving generality, but, more important, we almost always fail to assess validity of prior findings rigorously. Of course in nature so many variables are beyond the control of researchers that no study can ever be perfectly replicated, and in some cases, as with critically endangered species, replication may be infeasible and even unethical [12]. Additionally, long-term data sets cannot be replicated on a whim or with anything short of profound dedication [13]; though many long-term data sets can themselves be treated as a series of replications, and should be valued as such. So where does this leave us?

Although each ecological and evolutionary study is unique and thus strictly speaking cannot be proven wrong through replication, this certainly does not make replication to assess validity of an earlier finding a waste of time. Across disciplines, there is a continuum of possible precision in replications, and perfect exact replication (see definitions below) may typically be unattainable in any discipline. Thus, we should develop an appropriate understanding of how replication can assess validity. When a replication fails to confirm an earlier finding (for example, red colour rings increase male zebra finch courtship behaviour), it may be true that conditions have changed and both studies were correct in their own contexts. This scenario, however, is no more plausible than the possibility that either the original study or the replication is incorrect. To build confidence in our understanding, we must conduct multiple robust replications and combine their results with quantitative research synthesis. The meta-analysis of zebra finch colour ring effects described above is an ideal example [5]. Quantitative syntheses will be best suited to assess validity of an earlier study when researchers have minimized differences in methods and environmental conditions, ideally while also systematically varying conditions hypothesized to influence the study outcome (see below; [14–16]). This systematic approach helps us build confidence in tests for validity while simultaneously initiating the process of circumscribing the generality of the phenomenon. We can never be certain that an aberrant study was incorrect. Yet, with enough robust contradictory evidence, we can treat it as incorrect or at least as insufficiently general to merit further consideration. To return to the zebra finch example, when four replications fail to find an effect of ring colour on male courtship rate and body mass, it becomes reasonable to discount the original claim that ring colour influences male zebra finches in these ways [5]. We address the goal of determining generality below.

## Scientific replication and generality

Traditionally, three main levels of replications are recognized; exact, partial and conceptual [10]. We follow Palmer [9] and include quasi-replication (cross-species or system), though we point out that quasi-replications can also vary from partial to conceptual. The first level, exact (sometimes also known as ‘direct’ [17]) replication, provides the highest fidelity to the original work. As mentioned above, given the complexities of the systems in ecology and evolutionary biology, exact replication is never perfectly attained and so attempts at exact replication are, at best, ‘close’ replications. Partial replications fall along a spectrum, from these ‘close’ replication to replications that include limited procedural or methodological differences. The four replications of the zebra finch colour ring work should be considered partial (but close) replications, despite the authors’ effort to replicate the original work precisely, because the breeding history of animals and the details of rearing conditions differed from those of the original study and among the replicates [5]. As explained above, however, differences were slight enough to allow us to reject the validity of the original finding based on the replications.

While a close replication attempts to duplicate experimental methods, a conceptual replication uses a distinctly different study designed to test the same hypothesis [17]. For instance, two studies presented reasonable tests of the hypothesis that male plumage colour in blue tits signals potential aggression towards conspecific males [18, 19], but they did so with dramatically different methods (captive versus wild, response to live threats versus taxidermic mounts, different measurements of aggression, and so on). If both of these studies had found that male plumage colour predicted male aggression, we would have gained confidence in the original hypothesis. As it is, one study supported this hypothesis and the other did not. Because these studies should be considered conceptual replicates, the differences in results between them could either mean that one of the studies was in error, or that both studies were correct in the context of their methods [6]. The latter case would mean that more narrowly defined hypotheses reflecting the set of conditions imposed by each experiment could be true even if the original, more broadly defined, hypothesis described above were not true. These new hypotheses would, however, require further testing [6].

Because conceptual replications are defined simply as distinct tests of the same hypothesis, the definition of the hypothesis is the only factor constraining what we consider a conceptual replication. To continue with the blue tit example from the previous paragraph, we could redefine the hypothesis as ‘male plumage colour in blue tits is a signal used in conspecific interactions’, in which case we would also want to consider, for example, responses to this putative signal by females in studies designed to assess mate choice. Alternatively we could expand in other directions, for instance by hypothesizing that ‘male phenotypic traits signal potential aggression towards conspecific males’, thus allowing us to consider traits other than plumage colour.

We may wish to expand our scope beyond the original species or system with quasi-replication [9]. It could be useful to conduct a quasi-replication in which we match methods from the earlier study. This form of partial replication would allow any differences in results to be more confidently attributable to differences between species or systems rather than to different methods. However, most quasi-replications are conceptual in nature and make no effort to match methods. Thus, like conceptual replication within species, these replications can help define generality, but when results conflict, we cannot draw robust inferences about why this may be. Most large meta-analyses published today in evolutionary biology and ecology combine quasi and conceptual replications. These analyses thus help us determine generality of phenomena (within limits; see below), but without close replications within systems and species, these analyses are not effective at assessing validity of published findings. In Table 1, we summarise the relationship between levels of replication, assessment of validity, and establishment of scientific generality.

**Table 1:** The different levels of study replication in relation to establishing validity and generality

Replication level	Testing validity	Scope of generality
Exact (close) replication	Excellent	Narrowly defined biological phenomenon limited to a population, strain, or locality
Partial replication	Good	Fairly narrowly defined biological phenomenon mostly limited to a population, strain, or locality
Conceptual replication	Poor	Species- or system-specific phenomenon, broadly defined
Quasi-replication (partial)	Poor	Narrowly defined biological phenomenon across species or system
Quasi-replication (conceptual)	Poor	Broadly defined biological phenomenon across species or systems

Replications, regardless of level, should all be welcome. Quantitative synthesis of empirical studies (meta-analysis) relies on availability of many replications; the statistical toolboxes of meta-analysis are equipped to help identify potential selective reporting as well as generality [14–16]. As should be clear, individual replications entail a trade-off between levels of generality and tests for validity (Table 1). Thus, all levels are valuable in their own way and the choice of a level would seem to rest with the researcher’s goals. However, robust inferences about generality require sound inferences about validity so replications should ideally be performed in order starting with close replication and only later moving to conceptual and quasi-replication.

The desirability of first replicating at the exact (or more realistically, close) level has been recognized for some time. Palmer [9] laid out the case that quasi-replication, although useful, if used alone is not a robust method for a determining cross-species or cross-system generality. First, as we discussed above, no replication can definitely prove an earlier study wrong, but close or rigorous partial replication is often the only effective tool for identifying results that are likely wrong. Thus, a series of strictly quasi-replications (for example, across species or systems) cannot actually determine whether a hypothesis is supported or contradicted in any of the given species or systems included. Further, high-level replications (that is, quasi- and conceptual replications) are prone to misuse because, unlike exact or close replications, researchers can decide which data to analyse and what to report. Unfortunately, this kind of ‘researcher degrees of freedom’ [20] seems pervasive across many scientific fields [21]. Simonsohn and colleagues [22] recently termed the practice of selective analysis and reporting (researcher degrees of freedom) ‘*p*-hacking’, which is distinct from traditional publication bias (where non-significant results are less likely to be published) because *p*-hacking is a suite of potentially deliberate acts that bolster a researcher’s chance of finding significant *p* values. Thus, we expect a collection of quasi-replications to be a biased collection of studies that will produce a biased average effect in research synthesis. On the other hand, if the effect from each species or system derives from a series of close replications, we will have substantially more confidence in the patterns within each species or system, and thus substantially more confidence in the overall effect derived from cross-species (or system) synthesis.

However, the meaning of quasi-replication is not as clear as it first appears. Within a species, populations differ from each other in multiple ways. Just as with different results in different species, when results differ between two populations of the same species, we cannot distinguish real population differences from some sort of error. We do not claim to have a definitive answer to the conundrum of where to draw the boundaries of quasi-replication. However, we do have some thoughts on this matter. First, we repeat our assertion that the closer a replication conforms to the original study in methods and experimental conditions, the more valuable that replication is for assessing the validity of the original. Further, clear thinking about the scope of intended inference can help us determine what sort of replication is needed. If we are testing the generality of a phenomenon within a species, then among-population replication is useful and may be sufficient. If we simply wish to know whether the plumage of male blue tits reflects more light in the UV than does the plumage of female blue tits, regardless of population, then a series of different studies in different populations is fine. However, if we wish to draw robust conclusions about among-population differences, then we need to conduct replications within each of the populations of interest [6]. Some studies have found greater sexual dimorphism in blue tit plumage colour than have others [6], but we cannot attribute this difference to biological differences in populations since we lack sufficient replicates to assess the validity of these differences.

It is worth pausing here to acknowledge that not only might we expect results to vary geographically within a species, we also can expect results to vary from year to year due to real temporal variation in biological processes (for example, [23], but see [24]). Such variation presents serious challenges to replication [6]. However, if we find a result in one year and not in another, we cannot robustly infer temporal variation without replication. Often researchers understand their system well enough to expect different process between, for instance, wet and dry years. In this example, confirming differences in replicate wet and dry years will add substantial inferential strength. In some situations, replication within divergent conditions will be very difficult. Thus, we will have to accept a lower level of confidence in our inference.

Another reason to promote within-species or within-system replications is that although we value broad generality of research findings, we readily acknowledge that idiosyncratic or species-specific findings can have important implications. For instance, it appears unique that naked mole rats, *Heterocephalus glaber*, do not suffer from cancer. It is this idiosyncrasy that possibly carries a huge potential for cancer treatment [25]. Medical significance aside, population-specific adaptations of a particular species can be of great interest to evolutionary biologists as they often provide clues to a bigger question on the process of evolution.

Note that we have not provided a comprehensive analysis of replication above. There are many more challenges we have not explored and replication strategies that we have not explained. However, we have attempted to lay out the basic case in favour of replication.

Although all forms of replication have value, they are not all equally common. Various forms of conceptual and quasi replication are much more common than close or careful partial replications [9, 10]. Given the importance of close replication to building a foundation of understanding within species and systems, our highest priority right now is to shape incentive structures to promote systematic close replications.

## Initiatives for replicative and reproducible research

We recently reviewed initiatives in medical and social sciences to encourage replication and to discourage selective reporting such as publication bias and *p*-hacking; the former and the latter are intertwined as selective reporting influences reproducibility of research [26]. Four notable examples of initiatives in other disciplines are: (i) the development of a set of unified standards to increase reproducibility, taken up by many journals in biomedical sciences [27] and psychology [11], (ii) systematic efforts to coordinate, fund, and publish replications [28], (iii) the use of ‘registered reports’ to review proposed replications prior to gathering data and guarantee publication of accepted proposals independent of outcome [29], and (iv) non-profit organizations awarding ‘badges’ for reproducibility [29]. The field of ecology and evolution, as a whole, has yet to unite for similar initiatives.

Fifteen years ago, Palmer argued that three groups of people would need to come together to establish a culture of replications in ecology and evolution [9]. These groups are editors, funders, and supervisors (or principal investigators). However, it is important to remember as we discuss these that the first two groups play a major role in establishing the incentive structures that shape the choices of the third group, the principal investigators.

Journal editors can provide an incentive to replicate by creating journal sections dedicated to publishing replication work. As simple as this suggestion seems, no journals in ecology and evolution have had such a section until now. To our delight, the renowned evolutionary biologist John Endler is initiating a new section, named ‘New Tests of Existing Ideas’ in the journal *Evolutionary Ecology* [30]. What is more, the best paper published in this new section will be awarded a prize named after R.A. Fisher, a father of modern evolutionary biology and statistics and a strong believer in replicating experiments. We hope other ecological and evolutionary journals will follow this example. With many journals moving online, the old excuse of limited journal space should fade as an obstacle to publishing replicative research. The effectiveness of this incentive for replication will presumably be stronger as more journals, especially those with high impact factors, adopt replication sections.

As high status outlets for publishing excellent replications emerge, the idea of professors or institutions requiring some replication of previous work as a part of every PhD thesis becomes more plausible [9, 10]. It is eminently sensible to ask a student to replicate a key study that constitutes part of the foundation of a given PhD thesis. To make this scenario more common, support from PhD supervisors and universities will be essential, along with incentives from the broader scientific community. The possibility of publishing replications in high-impact journals is an ideal incentive for toiling away at the task. Like it or not, the value of scientists is often measured by publications in high-impact journals, especially for those who have yet to land a permanent job [31].

Equally important, replication needs funding, which in our field is awarded almost exclusively for novel scientific quests. It is funders, therefore, who can have the greatest and most immediate influence on our attitude towards replication. Researchers will scramble to replicate when money is set aside for this purpose [9]. Funding agencies could promote replication batteries through collaboration on simultaneous replications across different laboratories [3]. Such collaborative projects could be an effective path towards replication for ecologists and evolutionary biologists. Researchers could work together to test hypotheses on the same, closely or distantly related species in a standardized manner [32], ideally via a registered reports framework [28]. Another idea is for agencies to evaluate reproducibility of foundational studies as a criterion for awarding research funding [6] or to provide funds for replications of previous research as part of grants for novel work. Of course, replications may contradict previous work, in which case scientists would need to step back and further investigate foundational ideas. In so doing, they would avoid wasting time and money on projects resting on unsound foundations. Changing funding protocols may not be as easy as adding replication sections to journals. However, it is up to us to push funding agencies in this direction.

Allocating resources to robust replication presumably will divert resources from other projects. Some will argue that this diversion is foolish. Instead, we believe it is foolish not to invest in substantiating important studies. Failing to invest in replication means that too often we will instead invest in novel studies resting on flawed foundations, and that is clearly a poor investment. We invested in the original studies because they held the potential of answers worth knowing. If we really do want these answers, then we need replication.

Finally, we return to the question of whether evolutionary biologists and ecologists will be able to increase the rate of replication sufficiently to improve the quality of inferences in our disciplines. In theory, replication at any level should be extremely valuable as a fundamental part of cumulative science. The real questions are whether ecologists and evolutionary biologists as a community value replication and are willing to promote it despite the obvious obstacles. We hope that we can convince a sufficient number of our colleagues that the potential gains in the quality of scientific inference from replication are worth the costs. We will continue to work for shifts in editorial policies, graduate training programs and research funding priorities [9].
